# Changes in biomarkers of exposure and biomarkers of potential harm after 360 days in smokers who either continue to smoke, switch to a tobacco heating product or quit smoking

**DOI:** 10.1007/s11739-022-03062-1

**Published:** 2022-08-28

**Authors:** Nathan Gale, Michael McEwan, George Hardie, Christopher J. Proctor, James Murphy

**Affiliations:** 1 B.A.T. (Investments) Limited, Regents Park Road, Millbrook, Southampton, SO15 8TL UK; 2 DoctorProctorScience Limited, 157 Cavendish Meads, Sunninghill, Ascot, SL5 9TG UK

**Keywords:** Cigarette smoking, Tobacco heating product, Biomarkers of exposure, Biomarkers of potential harm, Modified risk tobacco product

## Abstract

The aim of this study was to investigate whether biomarkers of exposure (BoE) and potential harm (BoPH) are modified when smokers either continue to smoke or switch from smoking cigarettes to exclusive use of a tobacco heating product (THP) in an ambulatory setting over the period of a year, and to compare any changes with smokers who quit tobacco use completely and with never smokers’ biomarker levels. Participants in this year-long ambulatory study were healthy smokers with a self-reported low intent to quit assigned either to continue smoking or switch to a THP; a group of smokers with a self-reported high intent to quit who abstained from tobacco use; and a group of never smokers. Various BoE and BoPH related to oxidative stress, cardiovascular and respiratory diseases and cancer were assessed at baseline and up to 360 days. Substantial and sustained reductions in BoE levels were found at 360 days for both participants who switched from smoking to THP use and participants who quit smoking, in many cases the reductions being of a similar order for both groups. The never smoker group typically had lower levels of the measured BoEs than either of these groups, and much lower levels than participants who continued to smoke. Several BoPHs were found to change in a favourable direction (towards never smoker levels) over the year study for participants who completely switched to THP or quit, while BoPHs such as soluble intercellular adhesion molecule-1 were found to change in an unfavourable direction (away from never smoker levels) in participants who continued to smoke. Our findings, alongside chemical and toxicological studies undertaken on the THP used in this study, lead to the conclusion that smokers who would have otherwise continued to smoke and instead switch entirely to the use of this THP, will reduce their exposure to tobacco smoke toxicants and as a consequence are reasonably likely to reduce disease risks compared to those continuing to smoke.

**Supplementary Information:**

The online version contains supplementary material available at 10.1007/s11739-022-03062-1.

## Introduction

Combustible cigarette smoking is an important avoidable cause of a wide range of chronic disease including lung cancer, chronic obstructive pulmonary disease and cardiovascular diseases [1]. The risks of developing smoking-related disease vary by disease but generally increase with increasing exposure through the number of years of smoking and daily cigarette consumption [2, 3]. Cigarette smoking causes dependence, mainly because of the effects of nicotine, but most of the disease risk from smoking is thought to be related to persistent exposure to smoke toxicants other than nicotine, many of which are formed in the process of combustion of tobacco [1].

Epidemiology has shown that the excess health risks for smoking-related diseases reduce on smoking cessation, though the speed of such reductions vary by disease, by smoking history and by other factors including ethnicity and genetics [2, 3].

Public health approaches for decades have focused on preventing smoking initiation and increasing smoking cessation through a variety of measures, and many countries have adopted the World Health Organisation’s Framework Convention on Tobacco Control (WHO FCTC). In a recent review [4], it was noted that with 181 Parties to the Convention, articles on protection from exposure to tobacco smoke, packaging and labelling of tobacco products, education, communication, training and public awareness and sales to and by minors seem to have been implemented most successfully.

In 2000, the US Food and Drug Administration (US FDA) commissioned a report from the then US Institute of Medicine which looked at the scientific basis for tobacco harm reduction, looking at what scientific evidence might be needed to establish whether new tobacco and nicotine products could play a part in reducing the harm of tobacco use. This report proposed that new products with substantially reduced levels of toxicants in their emissions as compared to conventional cigarettes might provide less risky alternatives for smokers who would not otherwise quit smoking if they were satisfactory replacements for conventional cigarettes; that a new product’s reduced risk profile could be established through scientific evidence; and, that regulatory approaches should consider whether the introduction of new products would protect population health and not just the health of smokers [5, 6]. Several frameworks have been published that look at the types of chemical, toxicological and clinical studies that should be undertaken to assess the reduced risk potential of new products [7, 8].

With advances in micro-electronics two categories of potentially reduced risk tobacco and nicotine products have emerged over the past decade—tobacco heating products (THPs), and vaping products also known as electronic nicotine delivery systems (ENDS). Both categories use the ritualistic habit of smokers of bringing the product to the mouth, inhaling the emissions and delivering nicotine. Vaping products typically do not use tobacco, but rather high purity nicotine added to glycerol and/or propylene glycol to create an aerosol through a coil that is electronically heated [9]. THPs, sometimes described as Heated Tobacco Products or Heat not Burn Products, use battery powered electronics to heat tobacco to less than 350 °C, avoiding combustion and the formation of many of the toxicants found in cigarette smoke while still releasing nicotine and generating an aerosol with the addition of glycerol to the tobacco [9, 10].

The WHO’s Study Group on Tobacco Product Regulation (TobReg) reviewed published data on both vaping and THPs [10] and noted that much of the published literature on THP had been funded and conducted by manufacturers of the products, highlighting the importance of critically reviewing such data. Public health reports, for example, from Public Health England, have called for more data to be published on THPs [9]. A reasonable amount of chemical and toxicological research has been published on THPs, but most of the clinical data on THPs has focused on short-term exposures and changes in biomarkers of exposure (BoEs). Two 6-month studies have been published that report data on both BoEs and biomarkers of potential harm (BoPH) [11–13], and one 12-month study has looked at the potential impact of smokers’ switching to THPs on cardiovascular health indicators [14].

In this paper, we report data on a long-term (12 month) ambulatory clinical study that measured levels of BoEs and BoPHs in a UK-based population with groups of cigarette smokers that either continued to smoke, switched to a THP (glo™, often named THP1.1(RT) in the scientific literature) from a single manufacturer, or quit tobacco use completely (with optional support of nicotine replacement therapy and/or varenicline and cessation counselling). A group of never smokers were also recruited for comparative measures of BoEs and BoPHs.

Two short-term randomised clinical studies have already been published, one in Japan [15] and one in the UK [16], where participants were confined in clinic for around a week and randomised into groups that either continued to smoke, quit, or switched to a THP similar to that investigated in this study. These two prior studies showed that for BoEs for toxicants not formed or formed to a much-reduced amount in the THP emissions as compared to cigarette smoke (including carbon monoxide (CO), 1,3-butadiene, benzene, acrolein, crotonaldehyde, acrylonitrile, *o*-toluidine (*o*-Tol), 4-aminobiphenyl (4-ABP), 2-aminonaphthalene (2-AN) and ethylene oxide), reductions at end of study compared to baseline were statistically significant and of a similar amount for both the switch to THP and cessation groups. BoEs for two tobacco-specific nitrosamines (TSNAs), 4-(methylnitrosamino)-1-(3-pyridyl)-1-butanone (NNK) and N-nitrosonornicotine (NNN), were also measured. For these two BoEs reduction in levels at end of study compared to baseline were found in the switch to THP group but not to the same extent as in the cessation group.

The data reported in this paper are the final timepoint (at 360 days) of a pseudo-randomised, controlled, parallel group, open-label, ambulatory clinical study carried out at four sites in the UK (ISRCTN81075760). Data from the 90-day and 180-day timepoints have been published [13, 17] and showed sustained reductions compared to baseline in many of the BoEs at both timepoints for the group that switched to the THP and the cessation group, consistent with the short-term studies.

Longer term studies are necessary to observe any potential changes in BoPH because of the nature of most of these biomarkers. Most BoPHs used in this study are generic measures of biological pathways that are related to smoking-related diseases, but are not specific to tobacco use and will be influenced by a variety of lifestyle and genetic factors. At 180 days, several of the BoPHs had moved in a favourable direction for both the switch to THP and cessation groups, with the continue to smoke group either staying constant or moving in an unfavourable direction [13]. It should be noted that we use the terms favourable and unfavourable—these are subjective terms and simply mean that for a favourable change the BoPH level has moved towards that seen in the never smoker group and an unfavourable change is where the BoPH level has moved away from that found in the never smoker group.

Analysis of the 360-day timepoint data was performed as set out in the previously published statistical analysis plan, and statistical significance analyses were undertaken at the 180-day time point [13, 18]. Any primary endpoints found to be statistically significant at day 90 or 180 were not assessed in subsequent timepoints, with the assigned α level equally distributed between the remaining endpoints [19]. Because so many of the BoEs were statistically significantly different between baseline and 180 days in the two randomised groups (continue to smoke and switch to THPs) [13], the analysis of the 360-day dataset focuses on descriptive analysis between the four groups (continue to smoke, switch to THP, cessation and never smoke) and on any changes to BoPH. This analysis provides insights not only as to the impact of continuing to smoke, switching to a THP and cessation on BoEs and BoPHs, but also on the design and conduct of future studies.

## Methods

### Study design

This was a pseudo-randomised, controlled, parallel group, open label, ambulatory clinical study carried out at four sites in the UK (Belfast, London, Leeds and Merthyr Tydfil). Favourable opinion (which is equivalent to Institutional Review Board (IRB) approval) was given by the NHS Health Research Authority, Wales Research Ethics Committee 2 (reference number 17/WA/0212). The study was conducted in compliance with the ethical principles of the Declaration of Helsinki, Good Clinical Practice (International Council for Harmonisation (ICH) E6 Consolidated Guidance, April 1996) and UK laws, including those relating to the protection of participants’ personal data. Written informed consent was obtained from all individuals prior to their participation in the study and before undergoing any study procedures, including screening assessments. A full description of the study design, protocol and statistical plan has been published previously [18, 19]. This study is registered with ISRCTN (ISRCTN81075760).

### Participants

Eligible participants were healthy male or female adult current smokers (self-reported daily smoking of 10–30 non-menthol factory-manufactured or roll-your-own cigarettes for at least five consecutive years) or never smokers, all aged 23‒55 years. Regular smoking status was assessed using urinary cotinine (> 200 ng/mL) and exhaled breath CO (≥ 7 ppm). Inclusion and exclusion criteria have been described previously [18]. Participants who were never smokers or were planning to quit in the next 12 months were eligible only for the never smoker or cessation groups, respectively.

### Study procedures and randomisation

At Visit 1 (baseline), participants underwent safety and eligibility assessments prior to randomisation. Ambulatory 24-h urine samples and spot blood samples were taken for BoE and BoPH analysis, breath CO and fractional concentration of exhaled nitric oxide (FeNO) were measured, and spirometry was performed.

All participants except the never smoker group attended the clinic on days 30, 60, 90, 180, 270 and 360, at which the same samples were collected as Visit 1. The never smoker group attended on days 90, 180 and 360.

Adverse and serious adverse events were monitored throughout the study period by open questioning at each study visit and by encouraging participants to spontaneously report such events by telephone should they occur between study visits. Reported adverse events were recorded in source data and on electronic case report forms and coded according to MedDRA Version 20.0.

### Investigational products

Participants in the continue to smoke group (A) were required to purchase their own usual-brand cigarettes. Those in the switch to THP group (B) received the glo™ THP device and neo stick tobacco consumables (B.A.T. (Investments) Limited, Southampton, UK) free of charge. These products have been described previously and consist of a rechargeable electronic device that is used to heat in a controlled manner a disposable stick consisting of processed tobacco contained in paper to a maximum temperature of 245 °C [20, 21]. Aerosol emissions profiles for the specific product batches used in this study have been reported previously [17].

At the beginning of the study participants randomised to Group B were provided by clinic staff with the THP device and tobacco consumables equivalent to 150% of their average number of cigarettes consumed per day (CPD) as self-reported at screening, with the possibility of obtaining more (up to a total of 200% of original CPD consumption) before visit 2 by visiting the study site. At visits 2–12, product usage was assessed by return of all empty, part-used and unused packs of THP consumables, and the next allocation of consumables was supplied at 120% of the usage in the previous period, up to the limit of 200% of pre-screening consumption.

For the cessation group (D) participants devised a cessation strategy with the Investigator, which included nicotine replacement therapy (NRT) and/or varenicline provision if requested, alongside cessation counselling.

A full statistical analysis plan including power calculation methods has been published previously [19]. Based on the power calculation, 466 smokers in total were enrolled, with the objective of having a minimum of 50 participants complete the study in full (i.e. through to day 360, with no major protocol deviations) in each of Groups A, B (CEVal compliant) and D. 40 never smokers were also enrolled with the aim of 30 such participants completing the study, since this was considered sufficient to characterise a never smoker benchmark.

### Compliance

Participants were instructed of the importance of exclusively using their randomised product (Groups A and B) or of not smoking cigarettes or using nicotine products (Groups D and E) other than NRT (Group D). Compliance to protocol is a challenge for these types of studies because smoking behaviours can be resistant to change. All participants in this study other than the never smoker group were regular smokers on recruitment, and lack of compliance in the switch to THP and cessation groups through occasional or frequent smoking between clinic visits would affect both BoE and BoPH levels.

This was tackled in a variety of ways. The study design, in separating the recruitment of the continue to use tobacco groups to individuals declaring a low interest in quitting (and then randomising them to continue to smoke and switch to THP groups) from the cessation group, which was recruited from individuals with a high intent to quit, was created both for reasons of ethics and to help increase compliance. Similarly, providing the switch to THP group with free products and providing the cessation group with cessation treatments and counselling was intended to increase compliance. Managers of the clinics emphasised the importance of compliance to participants, who maintained electronic diaries on product use. Participants who were self-declared non-compliant were asked to leave the study. A long-term marker of compliance, N-(2-cyanoethyl)valine (CEVal), a haemoglobin adduct of acrylonitrile which is a toxicant relatively specific to cigarette smoking (though other environmental sources do exist) was used post study to analyse fractions of the completing participants more likely to have been completely compliant to protocol [13, 17, 22]. Using thresholds calculated based on a previous study [22], participants with CEVal levels < 78 pmol/g globin, < 54 pmol/g globin, and < 35 pmol/g globin at days 90, 180 and 360, respectively, were considered more likely to be compliant with the restriction to not smoke cigarettes (“CEVal compliant”).

### Biomarkers of exposure

BoE to selected cigarette smoke constituents in 24-h urine collections were measured at baseline and days 30, 60, 90, 180, 270 and 360. Laboratory analyses of urine and blood BoE were carried out at ABF GmbH (Planegg, Germany). Details of the bioanalytical methods have been published previously [13, 17].

BoE measured in 24-h urine samples were total nicotine equivalents (TNeq; nicotine, cotinine, 3-hydroxycotinine and their glucuronide conjugates); total 4-(methylnitrosamino)-1-(3-pyridyl)-1-butanol (NNAL); total NNN; 3-hydroxypropylmercapturic acid (3-HPMA); 3-hydroxy-1-methylpropylmercapturic acid (HMPMA); S-phenylmercapturic acid (S-PMA); monohydroxybutenyl-mercapturic acid (MHBMA); 2-cyanoethylmercapturic acid (CEMA); 4-ABP; *o-*Tol; 2-AN; 1-hydroxypyrene (1-OHP); and 2-hydroxyethylmercapturic acid (HEMA). Additionally, CO in exhaled breath and CEVal in whole blood were measured.

### Biomarkers of potential harm

BoPH were assessed in urine (11-dehydrothromboxane B2 [11-dTx B2], 8-epi-Prostaglandin F2a type III [8-Epi-PGF2α type III]), whole blood (white blood cell [WBC] count), plasma (soluble intercellular adhesion molecule-1 [sICAM-1]), serum (high-density lipoprotein [HDL]) and exhaled breath (FeNO). Additionally, forced expiratory volume in 1 s (FEV_1_) was assessed using spirometry. Indications associated with each BoPH have been reported previously [18, 19]. While NNAL is generally used as a BoE to the cigarette smoke toxicant NNK, it is also considered to be a BoPH for smoking-related lung cancer risk due to its tobacco specificity, its carcinogenicity, and its predictive value for lung cancer risk [23–25]. Laboratory analyses of urine and blood (whole, plasma and serum) BoPH were carried out at Celerion (Lincoln, NE, USA) and Covance (Harrogate, UK and Geneva, Switzerland). Details of the methods used for analysis of BoPH have been reported previously [13].

## Results

### Participant demographics

The first participant was enrolled onto the study on 7th March 2018, and recruitment was completed on 31st March 2019. Of smokers with no intent to quit, 79 were randomised to Group A and 197 to Group B, and 190 smokers intending to quit were enrolled into Group D. Of these, 21 in Group A, 81 in Group B, and 90 in Group D were withdrawn before or missed their day 360 visit. Three participants in Group A, none in Group B, and 4 in Group D had major protocol deviations affecting that visit. Thus, 55, 116 and 96, respectively, were included in the day 360 per protocol population.

40 never smokers were enrolled into Group E; 6 of these participants withdrew from the study prior to the day 360 visit and as such 34 were included in the day 360 per protocol population.

Brief demographic details for participants in all groups are presented in Table 1.

**Table 1:** Demographic data for study participants in the day 360 per protocol population

		Group			
		A (continue to smoke)	B (switch to THP)	D (cessation)	E (never smokers)
*N* (PP population)	*N*	55	116	96	34
Age (years)	Mean (SD)	38 ± 10.1	39 ± 8.5	39 ± 9.3	40 ± 10.0
Sex	Male:Female	30:25	63:53	57:39	13:21
Weight (males; kg)	Mean (SD)	81.0 ± 11.28	81.8 ± 11.03	82.7 ± 11.17	79.4 ± 12.41
Weight (females; kg)	Mean (SD)	66.3 ± 13.18	68.1 ± 9.85	66.8 ± 9.92	66.2 ± 8.70
BMI (kg/m^2^)	Mean (SD)	25.6 ± 3.68	25.6 ± 3.30	25.7 ± 3.05	25.4 ± 3.13
FTCD total score	Mean (SD)	5 ± 1.9	6 ± 1.8	5 ± 1.8	N/A
Cigarettes per day^a^	Mean (SD)	18 ± 5.6	18 ± 5.2	18 ± 5.3	N/A
Race					
Asian	*N* (%)	4 (7.3%)	7 (6.0%)	3 (3.1%)	2 (5.9%)
Black/African American	*N* (%)	3 (5.5%)	2 (1.7%)	3 (3.1%)	2 (5.9%)
White	*N* (%)	47 (85.5%)	104 (89.7%)	85 (88.5%)	30 (88.2%)
Other	*N* (%)	1 (1.8%)	3 (2.6%)	5 (5.2%)	0 (0%)
Ethnicity					
Hispanic/Latino	*N* (%)	0 (0%)	2 (1.7%)	0 (0%)	0 (0%)
Not Hispanic/Latino	*N* (%)	55 (100%)	114 (98.3%)	96 (100%)	34 (100%)

Data are as collected at screening

*BMI* body mass index, *N* number of participants, *PP* per protocol, *SD* standard deviation, *FTCD* Fagerström test for cigarette dependence, *N/A* not applicable

^a^Self-reported cigarette consumption at screening

### Cigarette and neo stick consumption

In Group A, self-reported cigarette consumption at all timepoints up to day 360 remained largely similar to that reported at screening (Table 2). In Group B, consumption of neo sticks was slightly higher than usual-brand combustible cigarette consumption reported at screening and in Group A at all timepoints but remained stable over time to day 360 (Table 2).

**Table 2:** Consumption data for study participants in the day 360 per protocol population

	Group A (continue to smoke)				Group B (switch to THP^a^)				Group D (cessation)			
	Baseline	Day 90	Day 180	Day 360	Baseline	Day 90	Day 180	Day 360	Baseline	Day 90	Day 180	Day 360
CC consumption^b^												
Number of participants	55	54	53	48	116	111	112	106	96	89	92	78
Mean (SD)	18.2 ± 5.6	17.8 ± 5.5	17.5 ± 4.7	16.8 ± 4.9	17.9 ± 5.2	0.0 ± 0.1	0.0 ± 0.1	0.0 ± 0.1	18.2 ± 5.3	0.1 ± 0.7	0.0 ± 0.1	0.0 ± 0.0
Minimum	10.0	7.4	8.7	8.8	10.0	0.0	0.0	0.0	10.0	0.0	0.0	0.0
Maximum	30.0	30.0	27.8	30.5	30.0	0.6	0.7	0.9	30.0	6.7	0.7	0.0
Neo stick consumption^c^												
Number of participants	–	–	–	–	–	113	113	115	–	–	–	–
Mean (SD)	–	–	–	–	–	20.9 ± 9.2	22.5 ± 9.6	24.6 ± 12.7	–	–	–	–
Minimum	–	–	–	–	–	0.7	0.4	3.8	–	–	–	–
Maximum	–	–	–	–	–	53.5	53.8	79.5	–	–	–	–

Data were recorded at ± 3 days up to day 90 or ± 14 days after day 90 due to individual participant visit scheduling. For cigarette consumption, data were averaged using daily self-reported consumption across all days between the relevant study clinic visits. Baseline combustible cigarette consumption data were self-reported by participants at screening. For THP consumption, the number of neo sticks dispensed at a participant visit minus the number of sticks returned at the subsequent visit was divided by the number of days between the two visits

^*a*^*THP* tobacco heating product

^b^Average number conventional cigarettes (CC) smoked per day. Number of participants is not equal at each timepoint within a group, since some participants failed to self-report CC consumption data; all available data are included

^c^Average number of neo sticks used per day. Three participants missed their day 90 visit, three missed their day 180 visit, and one did not return their products at their day 360 visit, thus their consumption data could not be calculated for these visits; all available data are included

### Compliance

CEVal measurement indicated compliance in 75 (65%) of the 116 participants in Group B reaching day 360. When applied in the same way to Group D participants, CEVal levels would indicate compliance in 62 (65%) of the 96 participants in this group reaching day 360 with no major protocol deviations. Similar to the observations at baseline, day 90 and day 180, only two never smokers had CEVal concentrations at day 360 above the assay lower limit of quantification of 2 pmol/g globin; their concentrations were 3.3 and 4.0 pmol/g globin. For Group A, all participants had CEVal levels exceeding the pre-specified threshold, indicating continued smoking.

### Adverse events

Up to day 360, exposure period adverse events occurred in 370 participants, including nine serious adverse events considered unrelated to any study product. The most frequently reported adverse event was headache, and most adverse events were mild or moderate in severity.

### Biomarkers of exposure

Table 3 presents the data for 14 biomarkers of exposure at day 1 (baseline), day 180 and at day 360 for the continue to smoke, switch to the THP, cessation and never smoker groups in the per protocol (PP) populations not adjusted for CEVal.

**Table 3:** BoE at baseline (day 1), day 180 and day 360 in the per protocol populations

Biomarker (units)	Group	*N*	Day 1	*N*	Day 180	*N*	Day 360
Total NNAL (ng/24 h)	A	56	214.69 (173.85, 255.53)	56	221.94 (177.86, 266.02)	51	164.73 (123.76, 205.71)
	B	123	204.53 (176.35, 232.71)	116	116.32 (99.90, 132.75)	108	69.82 (59.06, 80.58)
	D	117	232.41 (203.92, 260.91)	107	59.09 (39.41, 78.78)	91	46.16 (26.21, 66.11)
	E	33	12.80 (7.61, 17.98)	33	11.54 (5.95, 17.13)	31	3.81 (1.65, 5.96)
Total NNN (ng/24 h)	A	56	10.24 (5.19, 15.30)	56	9.73 (6.35, 13.12)	51	14.51 (8.20, 20.83)
	B	122	9.68 (8.05, 11.31)^a^	114	6.08 (4.54, 7.63)^b^	108	7.77 (5.38, 10.17)
	D	116	10.70 (7.95, 13.45)^c^	106	2.94 (2.13, 3.76)^d^	91	5.33 (3.95, 6.71)
	E	33	1.44 (0.33, 2.54)	33	1.24 (0.60, 1.89)	31	1.98 (0.51, 3.46)
3-HPMA (μg/24 h)	A	56	1128.51 (978.86, 1278.16)	56	1160.45 (978.58, 1342.33)	51	1191.11 (990.37, 1391.84)
	B	123	1240.63 (1078.53, 1402.74)	116	439.90 (364.66, 515.15)	108	500.83 (389.56, 612.10)
	D	117	1232.16 (1096.14, 1368.17)	107	351.99 (275.49, 428.48)	91	373.45 (261.55, 485.35)
	E	33	288.50 (184.02, 392.98)	33	261.83 (166.22, 357.45)	31	199.54 (144.59, 254.48)
HMPMA (μg/24 h)	A	56	433.24 (376.23, 490.25)	56	376.12 (317.68, 434.55)	51	394.72 (329.23, 460.21)
	B	123	455.11 (409.93, 500.30)	116	132.47 (110.28, 154.66)	108	128.25 (100.59, 155.92)
	D	117	427.72 (385.27, 470.16)	107	106.62 (90.91, 122.33)	91	105.06 (83.41, 126.72)
	E	33	111.00 (73.71, 148.28)	33	81.55 (66.29, 96.82)	31	81.89 (49.13, 114.64)
MHBMA (μg/24 h)	A	56	4.05 (3.01, 5.10)	56	3.70 (2.46, 4.95)	51	4.74 (3.30, 6.19)
	B	123	4.02 (3.40, 4.64)	116	0.67 (0.44, 0.90)	108	0.94 (0.64, 1.24)
	D	117	3.85 (3.14, 4.56)	107	0.47 (0.28, 0.67)	91	0.75 (0.48, 1.03)
	E	33	0.09 (0.01, 0.16)	33	0.24 (0.17, 0.32)	31	0.30 (0.23, 0.38)
HEMA (μg/24 h)	A	56	10.17 (7.09, 13.25)	56	4.95 (3.79, 6.11)	51	5.44 (4.21, 6.67)
	B	123	9.11 (7.85, 10.37)	116	1.77 (1.41, 2.13)	108	2.15 (1.73, 2.57)
	D	117	10.97 (9.48, 12.47)	107	1.79 (1.40, 2.18)	91	2.02 (1.57, 2.48)
	E	33	4.75 (3.58, 5.92)	33	1.30 (0.98, 1.61)	31	1.29 (1.02, 1.57)
4-ABP (ng/24 h)	A	56	18.87 (16.40, 21.34)	56	16.95 (13.95, 19.94)	51	29.36 (23.53, 35.19)
	B	123	19.48 (16.99, 21.97)	116	5.06 (4.29, 5.82)	108	5.66 (4.32, 7.01)
	D	117	20.37 (18.27, 22.47)	107	5.12 (4.27, 5.98)	91	4.67 (3.29, 6.06)
	E	33	3.74 (3.09, 4.39)	33	3.25 (2.51, 4.00)	31	1.29 (0.90, 1.68)
2-AN (ng/24 h)	A	56	26.46 (22.53, 30.39)	56	25.76 (21.33, 30.19)	51	18.54 (15.54, 21.55)
	B	123	27.87 (24.33, 31.42)	116	5.90 (4.30, 7.50)	108	5.82 (4.96, 6.67)
	D	117	28.29 (25.03, 31.54)	107	4.35 (3.25, 5.45)	91	5.52 (4.55, 6.49)
	E	33	1.89 (1.48, 2.30)	33	2.27 (1.34, 3.20)	31	2.93 (2.30, 3.56)
*o*-Tol (ng/24 h)	A	56	209.58 (98.26, 320.90)	56	155.65 (128.53, 182.78)	50	170.49 (140.84, 200.15)^e^
	B	123	182.22 (147.36, 217.08)	116	64.43 (56.17, 72.70)	108	73.00 (64.26, 81.73)
	D	117	176.33 (157.40, 195.27)	106	73.63 (50.77, 96.48)^f^	90	72.23 (63.61, 80.85)^g^
	E	33	48.99 (40.16, 57.81)	33	40.97 (32.94, 49.00)	30	51.50 (36.39, 66.60)^h^
1-OHP (ng/24 h)	A	56	259.85 (213.29, 306.41)	56	326.41 (245.93, 406.88)	51	327.48 (236.05, 418.91)
	B	123	286.25 (231.38, 341.13)	115	161.20 (125.62, 196.78)^i^	105	165.44 (121.95, 208.93)
	D	117	378.32 (322.62, 434.03)	107	136.26 (109.83, 162.69)	91	151.41 (111.85, 190.97)
	E	33	116.41 (91.58, 141.25)	33	77.98 (64.08, 91.88)	30	73.92 (52.15, 95.69)
eCO (ppm)	A	62	11.1 (8.9, 13.2)	59	11.8 (9.7, 14.0)	54	14.1 (12.0, 16.2)
	B	140	10.0 (8.9, 11.1)	125	2.3 (1.6, 3.1)	110	2.3 (1.6, 2.9)
	D	136	11.5 (10.4, 12.7)	108	1.4 (1.1, 1.7)	95	1.6 (1.2, 1.9)
	E	37	0.9 (0.6, 1.1)	37	1.1 (0.9, 1.3)	33	0.8 (0.6, 1.0)
TNeq (mg/24 h)	A	56	17.80 (15.12, 20.47)	56	13.37 (11.22, 15.53)	51	14.37 (11.84, 16.90)
	B	123	17.92 (15.91, 19.94)	116	11.87 (10.62, 13.11)	108	12.13 (10.78, 13.48)
	D	117	14.85 (13.22, 16.49)	107	2.52 (1.53, 3.50)	91	2.99 (1.70, 4.29)
	E	33	0.03 (0.01, 0.04)	33	0.01 (0.00, 0.01)	31	0.01 (0.00, 0.02)
S-PMA (μg/24 h)	A	56	4.46 (3.35, 5.57)	56	4.08 (2.77, 5.39)	51	4.88 (3.43, 6.33)
	B	123	4.41 (3.82, 5.01)	116	0.73 (0.49, 0.96)	108	0.79 (0.53, 1.06)
	D	117	4.36 (3.75, 4.98)	107	0.56 (0.34, 0.77)	91	0.56 (0.33, 0.78)
	E	33	0.28 (0.20, 0.36)	33	0.19 (0.15, 0.23)	31	0.10 (0.08, 0.13)
CEMA (μg/24 h)	A	56	173.62 (150.07, 197.18)	56	182.81 (154.77, 210.86)	51	204.53 (171.70, 237.37)
	B	123	194.52 (172.57, 216.47)	116	31.27 (20.41, 42.13)	108	32.62 (21.86, 43.39)
	D	117	181.62 (160.63, 202.62)	107	23.86 (13.69, 34.03)	91	32.21 (17.72, 46.71)
	E	33	1.88 (1.59, 2.16)	33	1.78 (1.42, 2.14)	31	1.63 (0.96, 2.31)

Group A, continue to smoke combustible cigarettes; Group B, switch to THP; Group D, cessation; Group E, never smokers. Data are means (95% CI). Three separate per protocol groups were defined at day 90, 180 and 360

*N* number of participants, *NNAL* 4-(methylnitrosamino)-1-(3-pyridyl)-1-butanol, *NNN* N-nitrosonornicotine, *3-HPMA* 3-hydroxypropylmercapturic acid, *HMPMA* 3-hydroxy-1-methylpropylmercapturic acid, *MHBMA* monohydroxybutenyl-mercapturic acid, *HEMA* 2-hydroxyethylmercapturic acid, *4-ABP* 4-aminobiphenyl, *2-AN* 2-aminonaphthalene, *o-Tol* *o*-Toluidine, *1-OHP* 1-hydroxypyrene, *eCO* exhaled carbon monoxide, *TNeq* total nicotine equivalents (nicotine, cotinine, 3-hydroxycotinine and their glucuronide conjugates), *S-PMA* S-phenylmercapturic acid, *CEMA* 2-cyanoethylmercapturic acid

^a^One outlier with a value of 1,015.16 ng/24 h was removed

^b^Two outliers with values of 636.06 and 456.26 ng/24 h were removed

^c^One outlier with a value of 349.72 ng/24 h was removed

^d^One outlier with a value of 297.65 ng/24 h was removed

^e^One outlier with a value of 37,961 ng/24 h was removed

^f^One outlier with a value of 16,989 ng/24 h was removed

^g^One outlier with a value of 5,583 ng/24 h was removed

^h^One outlier with a value of 4,828 ng/24 h was removed

^**i**^One outlier with a value of 27,972 ng/24 h was removed

Analytical chemical studies show that THP emissions for 1,3-butadiene, benzene, acrolein and CO are all reduced greater than 99% compared to cigarette smoke emissions from a reference cigarette [21]. Table 3 shows that the BoE for 1,3-butadiene (MHBMA) is low in never smokers (0.30 µg/24 h at day 360) and much higher in those continuing to smoke (4.74 µg/24 h). MHBMA reduces from baseline in both the switch to THP and cessation groups by similar amounts, though the day 360 levels are slightly higher (0.94 µg/24 h and 0.75 µg/24 h, respectively) than those found at day 180 (0.67 µg/24 h and 0.47 µg/24 h, respectively). When the data are adjusted to select only those participants that were both PP and CEVal compliant, then the 360-day MHBMA values drop to 0.42 µg/24 h (89% reduced from baseline) for the switch to THP group and 0.39 µg/24 h (89% reduced from baseline) for the cessation group, slightly higher than levels in the never smoker group.

For the BoE for benzene (S-PMA) never smoker levels are much lower (0.10 µg/24 h at day 360) than the continue to smoke group (4.88 µg/24 h). For the switch to THP and cessation groups, S-PMA is reduced from baseline to 0.73 µg/24 h at day 180 and 0.79 µg/24 h at day 360 for the switch to THP group, and to 0.56 µg/24 h at both day 180 and day 360 for the cessation group. For the CEVal-compliant participants the switch to THP group had a mean of 0.27 µg/24 h (93% reduced from baseline) and the cessation group 0.18 µg/24 h (96% reduced from baseline) at day 360.

Values for 3-HPMA, a BoE for acrolein, show a strong difference between the continue to smoke group (1191 µg/24 h at day 360) and the never smoker group (200 µg/24 h). The 3-HPMA means are slightly lower at day 180 compared to day 360 for both the switch to THP (440 µg/24 h compared to 501 µg/24 h) and the cessation group (352 µg/24 h compared to 373 µg/24 h). For the CEVal-compliant participants the switch to THP group had a mean 3-HPMA of 355 µg/24 h (68% reduced from baseline) and the cessation group 251 µg/24 h (78% reduced from baseline) at day 360.

Exhaled CO means showed large difference between smokers (14.1 ppm at day 360) and never smokers (0.8 ppm), and reductions from baseline in the switch to THP group (to 2.3 ppm) and cessation group (to 1.6 ppm) with little difference between day 180 and day 360. The CEVal compliant participants had CO means of 1.2 ppm (86% reduced from baseline) for those switching to THP and 1.0 ppm (91% reduced from baseline) for the cessation group.

BoEs for other toxicants reported in chemical studies as formed to a much-reduced amount in the THP as compared to cigarette smoke [21], including HMPMA (for crotonaldehyde), CEMA (acrylonitrile), HEMA (ethylene oxide), 4-ABP, *o*-Tol and 2-AN, reductions at day 360 compared to baseline were of a similar amount for both the switch to THP and cessation groups. Table 4 presents the percentage reductions compared to baseline values both for the whole of the PP population and for the subset of these that were also CEVal compliant. The percentage reductions at day 360 for the PP and CEVal compliant groups are greater in all but one case (1-OHP, which for the switch to THP group was a 42% reduction for the PP population and a 41% reduction for the PP and CEVal population) than those for the total PP group. The day 360 reductions from baseline across all the measured BoEs other than for TNeq, NNAL and NNN for the switch to THP group ranged from 42% to 83% for the whole PP group and 41% to 96% for the CEVal compliant group. For the cessation group the reductions in these analytes were 59% to 87% for the PP population and 62% to 97% for the PP and CEVal compliant population.

**Table 4:** Percentage reductions in BoEs from baseline to days 90, 180 and 360 for the per protocol and CEVal-compliant populations

Biomarker	Group	Day 90	Day 180	Day 360
Total NNAL	A	5%	+ 3%	23%
	B	55% (60%)	43% (47%)	66% (70%)
	D	83% (89%)	75% (89%)	80% (95%)
Total NNN	A	+ 29%	5%	+ 42%
	B	48% (52%)	37% (33%)	20% (26%)
	D	45% (68%)	73% (79%)	50% (65%)
3-HPMA	A	+ 10%	+ 3%	+ 6%
	B	62% (67%)	65% (67%)	60% (68%)
	D	72% (74%)	71% (72%)	70% (78%)
HMPMA	A	7%	13%	9%
	B	70% (74%)	71% (74%)	72% (79%)
	D	72% (72%)	75% (77%)	75% (81%)
MHBMA	A	+ 1%	9%	+ 17%
	B	76% (86%)	83% (90%)	77% (89%)
	D	90% (94%)	88% (94%)	81% (89%)
HEMA	A	20%	51%	47%
	B	43% (42%)	81% (84%)	76% (81%)
	D	30% (27%)	84% (87%)	82% (85%)
4-ABP	A	9%	10%	+ 56%
	B	69% (74%)	74% (78%)	71% (83%)
	D	73% (76%)	75% (80%)	77% (87%)
2-AN	A	4%	3%	30%
	B	77% (84%)	79% (85%)	79% (82%)
	D	85% (88%)	85% (90%)	80% (86%)
*o*-Tol	A	24%	26%	19%
	B	62% (64%)	65% (66%)	60% (63%)
	D	47% (44%)	58% (58%)	59% (63%)
1-OHP	A	+ 17%	+ 26%	+ 26%
	B	51% (52%)	44% (45%)	42% (41%)
	D	44% (56%)	64% (66%)	60% (62%)
eCO	A	+ 11%	+ 7%	+ 27%
	B	78% (83%)	77% (81%)	77% (86%)
	D	85% (86%)	88% (91%)	86% (91%)
TNeq	A	9%	25%	19%
	B	29% (31%)	34% (32%)	32% (29%)
	D	80% (86%)	83% (89%)	80% (91%)
S-PMA	A	13%	9%	+ 9%
	B	80% (89%)	83% (91%)	82% (93%)
	D	85% (92%)	87% (94%)	87% (96%)
CEMA	A	0%	+ 5%	+ 18%
	B	82% (90%)	84% (93%)	83% (96%)
	D	88% (95%)	87% (97%)	82% (97%)

Unbracketed values relate to baseline and day 90 values for participants who were in the day 90 per protocol population, day 180 values for participants who were in the day 180 per protocol population and day 360 values for participants who were in the day 360 per protocol population. Bracketed values for Groups B and D relate to baseline and day 90 values for participants who were in the day 90 per protocol population and were CEVal compliant at day 90, day 180 values for participants who were in the day 180 per protocol population and were CEVal compliant at day 180 and day 360 values for participants who were in the day 360 per protocol population and were CEVal compliant at day 360. In addition to the outliers noted in Table 3, one outlier in Group A for o-Tol at day 90 (41,329 ng/24 h) was removed prior to calculation of percentages

BoEs for two TSNAs, NNK (measured as NNAL) and NNN were also quantified. Both TSNAs are known to be present in THP emissions, but to a much lesser extent than in mainstream cigarette smoke. Reductions from baseline to 180 days for the BoEs for NNK and NNN were 38% to 43% for the switch to THP group and 73% to 75% in the cessation group. These were larger reductions for NNK than reported in the short-term studies for both switching to THP and for cessation groups, and similar reductions for NNN.

As presented in Table 3 levels of NNAL in the switch to THP and cessation groups were lower at day 360 than at day 180, with reductions from baseline (see Table 4) at 66% (70% for CEVal compliant) in the switch to THP group and 80% (95% for CEVal compliant) in the cessation group. The continue to smoke group also showed reductions compared to baseline of 23% at day 360. For NNN, BoE values were slightly higher for the switch to THP group at day 360 compared to day 180 and also higher for the cessation group, leading to reductions of 20% from baseline to day 360 for the switch to THP group and 50% for the cessation group. Never smoker levels of NNAL and NNN were lower than the continue to smoke, switch to THP, and quit groups at day 360.

Exposure to nicotine is measured by the BoE TNeq. At baseline the continue to smoke and switch to THP groups had similar TNeqs (17.8 mg/24 h and 17.9 mg/24 h, respectively), with the cessation group slightly lower at 14.9 mg/24 h. At day 360 the continue to smoke group had the highest mean TNeq (14.4 mg/24 h), with the switch to THP slightly lower (12.1 mg/24 h) and the cessation group much lower (3.0 mg/24 h). For the PP and CEVal compliant populations, the reductions from baseline to day 360 were 29% for the switch to THP group and 91% for the cessation group.

### Biomarkers of potential harm

The mean values for the BoPHs for the PP groups (and CEVal compliant for Groups B and D) are set out in Table 5. Included are the values for never smokers taken at day 360, as are the favourable direction of change. For example, for 11-dTx B2, which is related to platelet activation and coagulation, the favourable direction is a decrease towards never smoker levels and for FeNO, related to bronchodilation and vascular tone, the favourable direction is an increase towards never smoker levels [26].

**Table 5:** Descriptive statistics for BoPHs for the per protocol and CeVal compliant participant groups

BoPH	Group^a^	Favourable direction^b^/never smoker^c^	Day 1	Day 180	Day 360	Change from baseline^d^
8-epi-PGF2α (ng/24 h)	A	Decrease/176.46^e^	369.03	329.46	329.55	− 11%
	B		372.88	265.81	258.07	− 31%
	D		352.05	294.73	259.23	− 26%
11-dTx B2 (ng/24 h)	A	Decrease/676.45	1105.35	1030.97	1007.60	− 9%
	B		1101.51	827.77	890.06^f^	− 19%
	D		1302.56	969.44	919.17	− 29%
FeNO (ppb)	A	Increase/26.06	13.71	13.79	12.30	− 10%
	B		12.36	17.99	17.04	+ 38%
	D		11.13	22.65	21.23	+ 91%
WBC (× 10^9^/L)	A	Decrease/5.53	7.16	7.15	7.44	+ 4%
	B		7.63	6.42	6.25	− 18%
	D		6.91	6.29	6.14	− 11%
HDL (mmol/L)	A	Increase/1.73	1.39	1.37	1.46^ g^	+ 5%
	B		1.41	1.48	1.49	+ 6%
	D		1.56	1.58	1.54	− 1%
sICAM-1 (ng/mL)	A	Decrease/371.38	475.76	501.81	516.40	+ 9%
	B		464.36	405.82	427.90	− 8%
	D		411.71	433.23	391.38	− 5%
FEV_1_ (% pred)	A	Increase/97.23	91.50	88.07	86.22	− 6%
	B		91.85	93.04	92.14	0%
	D		93.35	92.40	93.75	0%

Data are day 1 and day 180 values for participants who were in the day 180 per protocol population and (for THP switch and cessation) were also CEVal compliant at day 180, and day 360 values for participants who were in the day 360 per protocol population and (for THP switch and cessation) were CEVal compliant at day 360

^a^Group A continue to smoke, Group B switch to THP, Group D cessation

^b^Direction of change thought to be favourable to health

^c^Mean value of the never smoker group at day 360

^d^Percentage change from baseline to day 360

^e^Outlier of 2761 ng/24 h removed

^f^Outlier of 6318 ng/24 h removed

^g^Outlier of 3.61 mmol/L removed

Comparing the data for never smokers with the baseline values for the continue to smoke group, switch to THP group and the cessation group, the never smoker values are in every case in a favourable direction. For several of the BoPHs the cessation group, which was recruited separately having a high intent to quit, are favourable compared to the continue to smoke and the switch to THP groups, who were recruited has having a low intent to quit.

In a previous paper [13], statistically significant differences were found between baseline and day 180 in the CEVal-compliant PP population for the group that switched to THP for 8-Epi-PGF2α type III levels and WBC count (which were reduced), and FeNO (which was elevated).

At day 360 8-Epi-PGF2α type III levels were similar for the continue to smoke group between day 180 and day 360 but were further reduced in the switch to THP and cessation groups. Change from baseline to day 360 was a 31% reduction for switch to THP and a 26% reduction in the cessation group, both trending towards but not reaching the never smoker levels.

For WBC the continue to smoke group had increased levels between day 180 and day 360, and the switch to THP group had reduced levels over the study period by 18% and by 11% in the cessation group.

The favourable direction for the breath BoPH FeNO is an increase and means reduced further in the continue to smoke group between day 180 and day 360, and reduced a little over this period for the switch to THP and the cessation groups but remained increased compared to baseline at a change of 38% for switch to THP and 91% for cessation, taking mean levels (17.0 ppb and 21.2 ppb) closer to those of never smokers (26.1 ppb).

11-dTx B2 reduced in the continue to smoke and cessation groups and increased slightly between day 180 and day 360 in switch to THP group, though remained decreased compared to baseline by 19% and 29% for switch to THP and cessation, respectively.

sICAM-1, associated with endothelial dysfunction, increased further between day 180 and day 360 in the group that continued to smoke, taking their mean value at 516 ng/ml further away from the never smoker mean of 371 ng/ml. The switch to THP group increased slightly from 406 ng/ml to 428 ng/ml between day 180 and day 360 while the cessation group reduced from 433 ng/ml to 391 ng/ml between these two timepoints. There was an overall reduction at day 360 from baseline of 8% for switch to THP and 5% for the cessation group.

FEV_1_ (% pred) continued to decrease in the continue to smoke group, giving a mean value of 86.2% at day 360, 6% reduced from baseline and much lower than the never smoker mean of 97.2%. Both switch to THP and cessation groups changed little over the year study period, with mean values at day 360 of 92.1% (from a mean baseline value of 91.9%) and 93.8% (from a mean baseline value of 93.4%) respectively.

HDL, associated with lipid metabolism, increased between day 180 and day 360 in the continue to smoke group by 6.6% and increased in the switch to THP group by 0.7%, but decreased in the cessation group by 2.5% between these time points. The cessation group had HDL levels closer to never smokers at baseline, and over the study period the slight decrease in their mean HDL remained closer to never smokers at day 360, followed by the switch to THP group and then continue to smoke group.

## Discussion

At 360 days, both mean levels of toxicant BoEs and mean levels of BoPH in never smokers illustrate the difference in smoking versus non-smoking status in terms of cigarette smoke related toxicant exposure and potential health risks. In all cases the BoEs were lower and the BoPHs more favourable than any of the other groups, even after 360 days of intervention for the switch to THP and cessation groups, though these two groups trended towards the never smoker values in most cases for both BoEs and BoPHs.

There were indications, particularly in the BoPHs, that the continue to smoke group had unfavourable changes in some BoPHs (FeNO, WBC, sICAM-1 and FEV_1_) through this year-long study.

Switching to THP resulted in sustained reductions through 360 days for all BoEs (to a lesser extent for nicotine than others) and the reductions tended to be slightly greater in the portion of the PP group that were measured to be CEVal compliant. This suggests that for those participants who were not judged to be compliant through the CEVal measure there may have been some occasional smoking occurring, but there was little to suggest that the PP group had significant regular smoking occurring. The same was true for the cessation group. For toxicants such as 1,3-butadiene and benzene that chemical studies suggest are not formed in THP emissions, the reductions from baseline increase from around 80% in the total PP group to around 90% in the CEVal portion of the group. This suggests that compliance was not a major issue in this study, and that some occasional smoking was not having a large effect on BoEs, but it would still be recommended that compliance biomarkers be used in such long-term studies. For most BoEs, reductions from baseline to day 360 were of a similar order for both the switch to THP and the cessation groups. This was not true for NNAL and NNN, where the switch to THP groups had reductions of 66% and 20%, respectively, compared to the cessation group with reductions of 80% and 50%, respectively, and was unsurprisingly not true of nicotine exposure with a 32% reduction in the switch to THP group and an 80% reduction in the cessation group. The fact that the NNN levels in the cessation group were only reduced by 50% (by 65% in the PP and CEVal compliant population) by day 360 of the study suggests that there might have been some analytical issues related to the measurement of this particular BoE, as even with nicotine replacement therapy interventions for some of the participants there is unlikely to be a significant source of NNN exposure for this group, and it would be expected over this time period that the compliant cessation group would have been close to never smoker levels of NNN. Artefactual, endogenous formation of NNN in the presence of nitrate/nitrite and nornicotine is possible [27–29] though, in our study, participants were screened as healthy thus not expected to have conditions leading to the acidic environment required in the bladder to catalyse this formation. It is more plausible that artefactual NNN may have been formed ex vivo, even under less acidic conditions, during storage of the urine samples prior to analysis [30].

For several BoPHs there were favourable directional changes over the study for both the switch to THP and cessation group that moved the levels of these BoPHs closer to the never smoker values, but in no case reached those levels. The epidemiology on smoking cessation suggests that rate of reductions in relative risk vary by smoking-related disease, smoking history and many other factors including genetics. Cardiovascular disease excess risks tend to reduce more quickly than lung disease risks [5], and in this study we found reasonably large changes from baseline to day 360 for BoPHs that can relate to CVD progression for the group that switched to THP including sICAM-1 (−8%), 11-dTx B2 (−19%), HDL (+ 6%) and FeNO (+ 38%). Markers for inflammation and oxidative stress showed favourable directional changes for the switch to THP groups (WBC −18%, 8-epi-PGF2a −31%) and in the cessation group (WBC −11%, 8-epi-PGF2a −26%). A BoPH related to lung heath, FEV_1_, showed no change over the study period for either the switch to THP or cessation groups, which may in part be due to the recruitment of healthy volunteers, though the continue to smoke group did move in an unfavourable direction.

None of the BoPHs showed an unfavourable change over the study period in the switch to THP and cessation groups, and while it is uncertain whether the favourable changes seen over this year period have measurable impacts on health risks, the changes in BoEs and BoPHs, added to what is known about the epidemiology of smoking cessation, leads to the conclusion that switchers to THP, if they maintain their behaviour are reasonably likely to reduce their relative risks for various smoking-related diseases as compared to continuing smokers.

There are relatively few long-term clinical studies on THPs. One reported 6-month study showed similar changes in BoEs and BoPHs with a different design of THP and in a population that was less compliant (i.e. had more off-protocol smoking) than the one reported here [12]. The WHO Study Group on Tobacco Product Regulation discussed this 6-month long study of 984 adult smokers in the USA [10]. The participants were randomised to switch to a THP (THS2.2) or to continue smoking, measuring changes in BoE and BoPH. Favourable changes in BoEs and in four BoPH (HDL, WBC, FEV_1_ and COHb) were reported in smokers who switched as compared with those who continued smoking. WHO TobReg reported that approximately 30% of smokers assigned to the THP became dual users of conventional cigarettes and THPs, but that in the group who predominantly used THPs the reductions in BoEs ranged from 16 to 49% of the baseline smoking level.

It is worth considering whether it was necessary to run this study for a full year. There were relatively small changes in both BoEs and BoPHs in either the switch to THP or the cessation groups between day 180 and day 360, and because of the way in which the statistical plan was set out, most of the formal statistical analysis was performed on the day 180 data. However, extending the study to a year gives much greater confidence in the sustainability of the BoE and BoPH changes and in the ability of the groups to maintain the conditions of not smoking and either switching to THP or quitting.

The study has several limitations. It was set up to longitudinally evaluate changes from baseline in a recruited group of healthy regular smokers, but only two groups (continue to smoke and switch to THP) could be randomised for both ethical (no intent to quit) and compliance reasons. The third group, cessation, was recruited from smokers with a high intent to quit which could have meant their behaviours related to smoking or their underlying health conditions (despite being judged to be healthy) might have been different to Groups A and B. This also meant that while statistical analyses could be undertaken between Groups A and B, it was not possible to undertake cross group analyses with Group D. The study was not powered to compare THP switch with cessation as, given the absence or large reduction in toxicants in THP emissions, it was expected that many thousands of participants would have been required to confirm equivalence or detect any statistically significant differences between the switch to THP and cessation groups.

While compliance was a focus and a long-term biomarker of compliance (CEVal) helped to assess compliance in the switch to THP and cessation groups, there was clearly some level of lack of compliance in the PP population. Moreover, and this may be related to some lack of compliance, BoEs were always lower in the never smokers than in any of the other groups. It might be expected that BoPHs might be directionally better in never smokers than in any of the other groups because of a history of regular smoking, but it is unknown whether the switch to THP or cessation groups would have eventually had BoEs and BoPHs similar to never smoking or whether there will always be residual levels of these markers, particularly with BoPHs.

That said, we believe that these data are convincing that switching to the study THP results in a sustained reduction in exposure to many tobacco smoke toxicants, that those reductions in most cases are similar to quitting tobacco use completely, and that both switching and quitting result in BoPHs moving closer to those found in never smokers.

In summary, this 12-month clinical study finds that regular smokers that either completely switch to a THP or quit smoking reduce their exposure to tobacco smoke toxicants substantially and sustainably, resulting in similar reductions in both groups to many tobacco smoke related BoEs and favourable changes to BoPHs. The continue to smoke group, even in this year-long study, showed sustained exposure to tobacco smoke toxicants and in some cases unfavourable changes in BoPH. The never smoking group always had lower BoEs and more favourable BoPH levels than any of the three groups that began the study as regular smokers.

The data support the public health view that not using tobacco is the safest choice and that smoking cessation leads to reductions in relative risks for smoking-related diseases, though this reduction may depend on smoking history and other factors. Our findings, alongside chemical and toxicological studies undertaken on the THP used in this study [21, 31–33], lead to the conclusion that smokers who would have otherwise continued to smoke and instead switch entirely to the use of this THP, will reduce their exposure to tobacco smoke toxicants and as a consequence would be reasonably likely to reduce their health risks compared to those continuing to smoke.

## Supplementary Information

Below is the link to the electronic supplementary material.

Supplementary file1 (DOCX 16 KB)

## Data Availability

The datasets generated during and/or analysed during the current study are available from the corresponding author on reasonable request.
