# Supplementary material for: Changes in biomarkers of exposure and biomarkers of potential harm after 360 days in smokers who either continue to smoke, switch to a tobacco heating product or quit smoking
Source: Intern Emerg Med. 2022 Aug 28;17(7):2017–30. doi: 10.1007/s11739-022-03062-1 (PMC9522838; doi:10.1007/s11739-022-03062-1)
Supplement: Supplementary file 1 — Supplementary file1 (DOCX 16 KB) [file 11739_2022_3062_MOESM1_ESM.docx]

Supplementary Table 1 Participant Disposition at Day 360

|  | **Group** | | | |
| --- | --- | --- | --- | --- |
|  | **A (continue to smoke)**  **N (%)** | **B (switch to THP)**  **N (%)** | **D (cessation)**  **N (%)** | **E (never smokers)**  **N (%)** |
| **Randomized/enrolled** | **79 (100%)** | **197 (100%)** | **190 (100%)** | **40 (100%)** |
| **Withdrawn** | **24 (30.4%)** | **81 (41.1%)** | **94 (49.5%)** | **6 (15.0%)** |
| Self-withdrew with no reason given | 7 (8.9%) | 28 (14.2%) | 35 (18.4%) | 3 (7.5%) |
| Lost to follow-up/not contactable | 4 (5.1%) | 14 (7.1%) | 5 (2.6%) | 1 (2.5%) |
| Adverse event/pregnancy | 1 (1.3%) | 1 (0.5%) | 3 (1.6%) | 1 (2.5%) |
| Positive drug screen | 8 (10.1%) | 22 (11.2%) | 15 (7.9%) | 0 (0.0%) |
| Resumed smoking | N/A | 8 (4.1%) | 11 (5.8%) | N/A |
| Missed crucial visit(s) | 0 (0.0%) | 2 (1.0%) | 9 (4.7%) | 1 (2.5%) |
| Other violation/non-compliance | 4 (5.1%) | 6 (3.0%) | 16 (8.4%) | 0 (0.0%) |
| **Included in Day 360 PP population** | **55 (69.6%)** | **116 (58.9%)** | **96 (50.5%)** | **34 (85.0%)** |

N, number of participants; PP, per protocol; N/A, not applicable. Percentages calculated using number of randomized/enrolled participants as denominator.
